# Toxicity Profile of eBAT, a Bispecific Ligand-Targeted Toxin Directed to EGFR and uPAR, in Mice and a Clinical Dog Model

**DOI:** 10.3390/toxins16090376

**Published:** 2024-08-26

**Authors:** Rose H. Dicovitsky, Jill T. Schappa, Ashley J. Schulte, Haeree P. Lang, Ellen Kuerbitz, Sarah Roberts, Taylor A. DePauw, Mitzi Lewellen, Amber L. Winter, Kathy Stuebner, Michelle Buettner, Kelly Reid, Kelly Bergsrud, Sara Pracht, Andrea Chehadeh, Caitlin Feiock, M. Gerard O’Sullivan, Tim Carlson, Alexandra R. Armstrong, Danielle Meritet, Michael S. Henson, Brenda J. Weigel, Jaime F. Modiano, Antonella Borgatti, Daniel A. Vallera

**Affiliations:** 1 Department of Veterinary Clinical Sciences, College of Veterinary Medicine, University of Minnesota, St. Paul, MN 55108, USA; dicov001@umn.edu (R.H.D.); scha0777@umn.edu (J.T.S.); ajgraef@umn.edu (A.J.S.); park2216@umn.edu (H.P.L.); ellen.kuerbitz@medvet.com (E.K.); sv.roberts6@gmail.com (S.R.); depa0049@umn.edu (T.A.D.); lewel001@umn.edu (M.L.); phil0467@umn.edu (C.F.); rolan048@umn.edu (A.R.A.); henso001@umn.edu (M.S.H.); modiano@umn.edu (J.F.M.); borgatti@umn.edu (A.B.); 2 Animal Cancer Care and Research Program, University of Minnesota, St. Paul, MN 55108, USA; alwinter@umn.edu (A.L.W.); stueb005@umn.edu (K.S.); buett005@umn.edu (M.B.); czap0013@umn.edu (K.R.); kbergsru@umn.edu (K.B.); prach011@umn.edu (S.P.); aleckert@umn.edu (A.C.); gos@umn.edu (M.G.O.); weige007@umn.edu (B.J.W.); 3 Experimental Surgical Services, Department of Surgery, Medical School, University of Minnesota, Minneapolis, MN 55455, USA; 4 Masonic Cancer Center, University of Minnesota, Minneapolis, MN 55455, USA; 5 Comparative Molecular Biosciences Graduate Program and DVM-PhD Dual Degree Program, College of Veterinary Medicine, University of Minnesota, St. Paul, MN 55108, USA; 6 Microbiology, Immunology, and Cancer Biology Graduate Program, Medical School, University of Minnesota, Minneapolis, MN 55455, USA; 7 Clinical Investigation Center, College of Veterinary Medicine, University of Minnesota, St. Paul, MN 55108, USA; 8 Department of Veterinary Population Medicine, College of Veterinary Medicine, University of Minnesota, St. Paul, MN 55108, USA; twcarlson2016@gmail.com; 9 Department of Population Health and Pathobiology, College of Veterinary Medicine, North Carolina State University, Raleigh, NC 27607, USA; 10 Department of Pediatrics, Medical School, University of Minnesota, Minneapolis, MN 55455, USA; 11 Department of Laboratory Medicine and Pathology, Medical School, University of Minnesota, Minneapolis, MN 55455, USA; 12 Center for Immunology, University of Minnesota, Minneapolis, MN 55455, USA; 13 Stem Cell Institute, University of Minnesota, Minneapolis, MN 55455, USA; 14 Institute for Engineering in Medicine, University of Minnesota, Minneapolis, MN 55455, USA; 15 Department of Radiation Oncology, Medical School, University of Minnesota, Minneapolis, MN 55455, USA

**Keywords:** targeted therapy, sarcoma, pharmacology, toxicity, immunotoxin

## Abstract

EGFR-targeted therapies are efficacious, but toxicity is common and can be severe. Urokinase type plasminogen activator receptor (uPAR)-targeted drugs are only emerging, so neither their efficacy nor toxicity is fully established. Recombinant eBAT was created by combining cytokines EGF and uPA on the same single-chain molecule with truncated *Pseudomonas* toxin. Its purpose was to simultaneously target tumors and their vasculature in the tumor microenvironment. In prior studies on mice and dogs, the drug proved efficacious. Here, we report the safety of eBAT in normal wildtype, uPAR knockout, and immunoreplete and immunodeficient tumor-bearing mice, as well as in dogs with spontaneous sarcoma that more closely mirror human cancer onset. In immunocompetent mice, tumor-bearing mice, uPAR knockout mice, and mice receiving species-optimized eBAT, toxicities were mild and self-limiting. Likewise, in dogs with life-threatening sarcoma given dosages found to be biologically active, eBAT was well tolerated. In mice receiving higher doses, eBAT was associated with dose-dependent evidence of liver injury, including portal biliary hyperplasia, oval cell proliferation, lymphoplasmacytic inflammation, periportal hepatocellular microvesicular change, hemorrhage, necrosis, and apoptosis. The results support continuing the clinical development of eBAT as a therapeutic agent for individuals with sarcoma and other cancers.

## 1. Introduction

Over the past several years, targeted cancer therapies have emerged as a new generation of cancer treatment. Targeted therapies interact with specific cell-surface receptors to inhibit growth and promote cell death. Targeted toxins or immunotoxins are a class of targeted therapies designed to deliver a potent catalytic toxin directly to the tumor site [1]. eBAT (epidermal growth factor-bispecific angiotoxin), a bispecific ligand-directed toxin [2,3], in a veterinary clinical study, was found to have efficacy against difficult to treat, and often fatal, spontaneously arising canine hemangiosarcomas [4]. Specifically, when added to standard of care surgery and chemotherapy, eBAT improved overall survival of dogs with stage 1/2 hemangiosarcoma by 65–70% (8.1 months at all doses and 8.6 months at the optimal dose vs. 4.9 months for a contemporary historical control treated with the standard of care). Moreover, the addition of eBAT to the standard of care increased the rate of long term survivors from approximately 7% (2/28 in the control group) to approximately 30% (5/17 in the dogs that received the biologically optimal dose of eBAT) [4].

eBAT is a recombinant bispecific ligand-directed toxin. DNA fragments encoding epidermal growth factor (EGF) and the amino acid terminal fragment (ATF) of urokinase type plasminogen activator (uPA) were spliced to DNA encoding truncated *Pseudomonas* exotoxin A (PE38) to produce a single-chain hybrid protein. The toxin PE38 has potent anticancer activity via inhibition of protein synthesis [5,6]. It was chosen because it was shown that delivery of less than 1000 molecules/cell was sufficient to cause complete tumor regressions [7]. To enhance potency, PE38 was modified by adding a Lys-Asp-Glu-Leu (KDEL) C-terminus signal to prevent secretion from the luminal endoplasmic reticulum [8]. The toxin was partially deimmunized via mutation of seven B-cell epitope-encoding sequences, identified by Onda and Pastan [9,10], to permit multiple in vivo treatments while minimizing anti-toxin immune responses. eBAT is designed to simultaneously target EGFR on solid tumors and uPAR on the vascular and inflammatory tumor microenvironment, thereby targeting cancer cells, as well as the cells that comprise the cancer niche to deliver a catalytic toxin, while minimizing effects on normal tissues [2,3].

Multiple EGF receptor (EGFR)-targeted therapies have been developed and used in patients with EGFR-overexpressing or EGFR-mutated tumors [reviewed in [11]. However, these drugs are commonly associated with significant toxicities consisting of, but not limited to, cutaneous toxicity, gastrointestinal toxicity, pulmonary toxicity, hepatotoxicity, cardiotoxicity, and nephrotoxicity [12,13,14,15,16,17,18,19,20]. Because eBAT targets EGFR, we hypothesized that it would have a similar toxicity profile as other EGFR-targeted drugs and designed studies to observe a large magnitude of change to test this hypothesis. Here specifically, we explored this hypothesis in studies measuring chemical, physiologic, and anatomical toxicities, and adverse events in eight studies where eBAT was administered systemically to healthy mice, tumor-bearing mice, and dogs with hemangiosarcoma enrolled in formal veterinary clinical trials. 

## 2. Results

### 2.1. The Effect of eBAT on Immunocompetent CD-1 Mice

**Figure 1:**
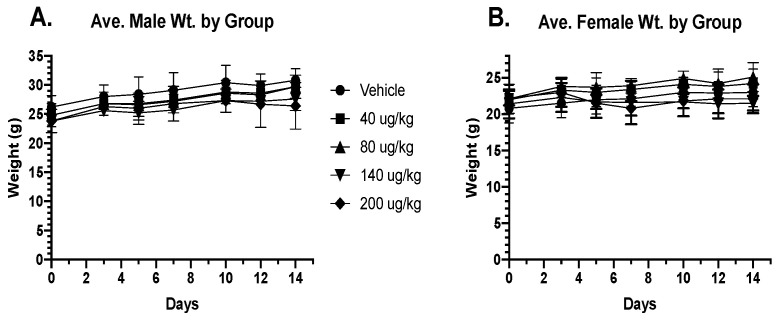
Weight in grams plotted over time in days for groups of male (**A**) and female (**B**) mice receiving increasing dosages of eBAT. Statistical analysis revealed no differences among any of the groups.

Previously, we observed that the bispecific ligand-targeted drug, eBAT, was safe in healthy B6 mice, whereas the corresponding monospecific toxins targeting EGF and uPAR were associated with severe toxicity, including dose-dependent lethality [4]. However, studies were purely observational and did not formally evaluate toxicity. Thus, we performed detailed experiments in mice and dogs to describe eBAT-related adverse events. In the first experiment evaluation of eBAT toxicity, four groups of twelve CD-1 mice (six male and six female) were administered increasing doses (40, 80, 140, and 200 µg/kg) of eBAT through the intravenous route in two weekly cycles (M, W, F), with one group of each sex receiving an equal volume of saline vehicle (0 µg/kg). Neither weight gain nor total body weight of either male or female mice was significantly altered (Figure 1A,B).

Hematological findings across all dose groups were clinically insignificant and thus interpreted to be minimally affected by eBAT administration. Mild abnormalities included lymphopenia, consistent with a stress/steroid leukogram; mildly increased neutrophil bands and rare neutrophil Döhle bodies, indicative of mild inflammation; and red blood cell morphology changes noted variably across all mice, including the control groups.

**Figure 2:**
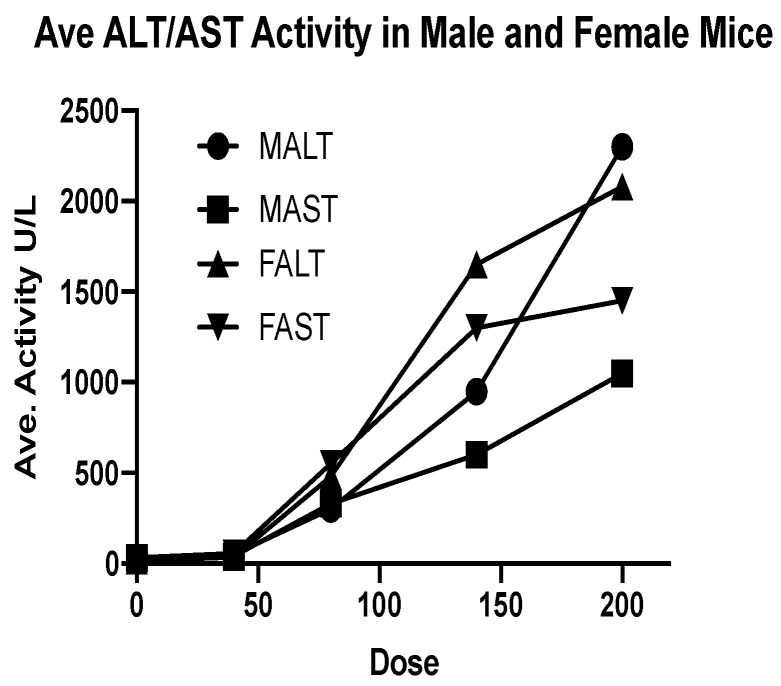
Dose-dependent changes in liver-specific enzymes in mice receiving eBAT. MALT: male ALT; MAST: male AST; FALT: female ALT; FAST: female AST.

The most significant findings noted on serum biochemistry panels were dose-dependent increases in hepatocellular damage enzymes, alanine aminotransferase (ALT), and aspartate aminotransferase (AST) (Figure 2). Liver function alterations included dose-dependent decreases in glucose and increases in bilirubin, especially at the two highest doses of 140 µg/kg and 200 µg/kg. There was also a dose-dependent trend towards hypoalbuminemia. 

Sporadic deviations from the reference interval were seen across all mice in creatine kinase values; various electrolytes including sodium, phosphorus, calcium, and potassium; and cholesterol. These sporadic deviations from the reference intervals were likely due to normal biologic variation or an imprecise reference interval for this mouse population. No clinically significant differences were noted between male and female mice. 

Most mice did not display adverse events that could be associated with dose administration, since 57 mice survived to study endpoint, corresponding to 95% overall survival. However, three early deaths occurred during the dosing phase of this experiment. One female mouse that received the highest dose (200 µg/kg) was found moribund and euthanized on study day 5 after the third of six doses was administered. Two other female mice receiving 200 µg/kg and 140 µg/kg, respectively, were euthanized due to >20% weight loss on day 9 after the fourth of six doses had been administered. 

Local toxicity included dose-related bruising at or near the injection site on the tails of two mice in the 140 µg/kg group and all of the mice in the 200 µg/kg dose group. There were no other clinical signs attributed to eBAT administration, indicating that mice tolerated eBAT well.

**Figure 3:**
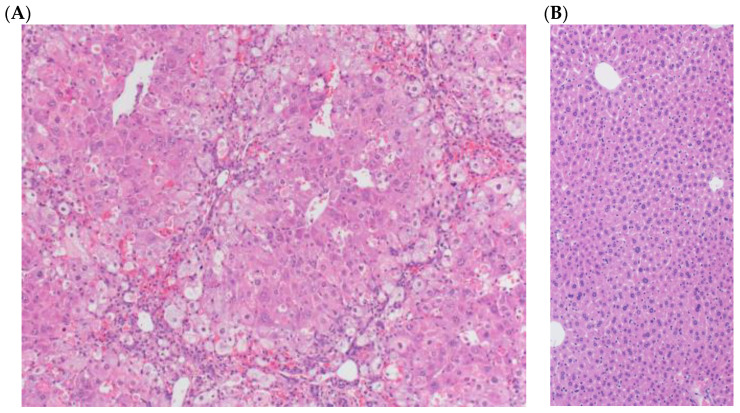
(**A**) Image of mouse liver histopathology from the 200 µg/kg (high dose) group, revealing extensive leukocyte infiltration, inflammation, and tissue damage. (**B**) Image of normal liver (100×).

Gross and histological assessment of organs at necropsy revealed toxicity was exclusively associated with changes in the liver at higher dosages. Liver histopathology results at experiment termination correlated with the enzyme studies. In mice receiving the two lower doses (40 and 80 µg/kg), there was evidence of mild to moderate periportal hepatocellular microvesicular vacuolar change and hepatocellular regeneration. Pathology in the mice receiving the two highest doses (140 and 200 µg/kg) consisted of dose-dependent portal biliary hyperplasia, oval cell proliferation, lymphoplasmacytic inflammation, periportal hepatocellular microvesicular change, hemorrhage, coagulation necrosis, and apoptosis (Figure 3).

In summary, adverse events were mostly limited to dose-dependent hepatocellular damage, which was corroborated by the histological changes in some of the mouse livers at necropsy. Spontaneous small intestinal villous atrophy and submucosal edema were seen in single cases and considered secondary or unrelated to eBAT. Major results from the mouse study are summarized in Table 1.

### 2.2. The Effect of Mouse eBAT (meBAT) on Immunocompetent Mice

The reduced level of eBAT-associated toxicity in normal immunocompetent mice might have been attributable to the relatively low affinity binding of cross-species uPA to its receptor on potential target cells. To rule out this possibility, we designed a mouse counterpart of eBAT, called meBAT. meBAT showed comparable cytotoxic activity and more robust binding to mouse cells than eBAT. But unlike human eBAT, meBAT had virtually no off-target activity against cells of canine origin. We evaluated systemic acute, subacute, and chronic toxicity of meBAT in immunocompetent mice. Three groups of four, six-week old, immunocompetent female and three groups of four male C57BL/6 (B6) mice were administered increasing doses (50, 100, and 200 µg/kg) of meBAT through the intraperitoneal route in two weekly cycles MWF, with one group receiving an equal volume of saline vehicle (0 µg/kg). The intraperitoneal route was chosen because it allowed for systemic distribution and biological activity, while decreasing the likelihood of local toxicity in and around the tail veins.

**Figure 4:**
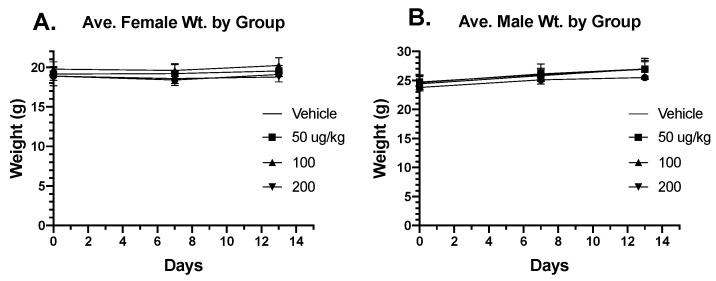
C57BL/6 mouse weight in female (**A**) and male (**B**) mice when given meBAT shows no differences compared to vehicle control. Statistical analysis revealed no significant differences.

No physical adverse events, including alterations in the rate of weight gain or total body weight (Figure 4) or postural or behavioral changes were noted in any of the mice during the experiment. For this and subsequent experiments using mice, we evaluated tissues histologically at the endpoint, minimizing risk of severe morbidity and premature mortality associated with longitudinal phlebotomy. We believe that this approach was appropriate and reliable to identify systemic toxicity: if we assume that adverse events occurred 10% of the time, with 24 subjects, there would be a 92% probability of observing at least one adverse event in the experiment, and if adverse events occurred 20% of the time, with 24 subjects, there would be a 99% probability of observing at least one adverse event in the experiment. Considering the relatively short exposure time of eBAT administration, direct assessment of organ toxicity provided a more robust measure of adverse events without having to rely on indirect or inferential biochemical testing. 

Gross necropsies were conducted at the experimental endpoint, and samples from liver, lungs, kidneys, adrenals, esophagus, heart, small intestine, pancreas, spleen, stomach, and omentum were examined microscopically in two representative animals from each group. Studies revealed hepatocellular vacuolation presumably from glycogen accumulation present across all treated groups. Although there were smaller numbers in this experiment compared to the more comprehensive CD-1 experiment, possible treatment-related hepatic effects were observed in one animal at 50 µg/kg that had a pale liver at necropsy and histological evidence of fatty change and presumably glycogen-related vacuolation, along with low-grade, mainly single-cell, hepatic necrosis. 

None of the B6 mice treated with eBAT or meBAT, or their organs, had features that were outside of the ordinary for the strain or species, except that eBAT and meBAT treatment in four and three animals, respectively, was associated with mild to marked increases in myelopoiesis in the liver and spleen. Myelopoiesis was more striking in meBAT-treated mice, possibly due to greater affinity of meBAT for mouse urokinase receptors, but this increase in myelopoiesis was not associated with signs of systemic inflammation or with other clinical signs of ill health in mice from either group.

### 2.3. The Effect of eBAT and meBAT on Tumor-Bearing BNX Mice

To determine if results were different in tumor-bearing mice, eBAT or meBAT was administered to BNX mice bearing tumors initiated with canine DHSA-1426 hemangiosarcoma cells [21]. Three groups of four BNX mice each were injected subcutaneously in the left flank with 2.5 × 10^6^ DHSA-1426 cells suspended in Matrigel (SF3). Three days later, the mice were treated with vehicle (group 1), eBAT (50 µg/kg, group 2), or meBAT (50 µg/kg, group 3) for a single weekly cycle (MWF), with no additional interventions until the experimental endpoint at day 40.

The severity of side effects associated with eBAT or meBAT administration to mice was not obviously higher when the mice had no immune system or when they harbored vascular tumors. Supplemental Figure S1 shows no significant change in weight following treatment. Toxicity in the BNX mice was evaluated based on gross changes in the appearance of skin, musculature, subcutaneous and visceral fat, lymph nodes, and visceral organs, as well as by histological parameters in liver, lungs, kidneys, adrenal glands, esophagus, heart, small intestine, pancreas, spleen, stomach, and omentum. Although two of the four mice in the control group and one of the four mice each in the eBAT and meBAT treatment groups died prematurely, the histological findings were not vastly different than those observed in the CD-1 and B6 experiments described above. Specifically, no remarkable gross or histological changes attributable to eBAT or meBAT treatment were seen in these animals (as above, none of the mice or the organs examined had features that were outside of the ordinary for the strain or species). The adverse events noted were directly attributable to the implanted tumors, which led to massive acute hemorrhage in the animals that died before the experimental endpoint. This clinical feature is often associated with rupture of devitalized tumor vessels as well as with coagulopathies caused by repeated microhemorrhagic events in humans with angiosarcoma and in dogs with hemangiosarcoma [22,23,24,25,26,27]. However, treatment-related toxicity cannot be completely ruled out as complicating factor.

### 2.4. The Effect of eBAT and meBAT on uPAR Knockout Tumor-Bearing B6 Mice

Having documented no adverse events from eBAT or meBAT in immunodeficient, tumor-bearing mice, we next evaluated the potential toxicity associated with these drugs in the setting of tumor-bearing immunocompetent wildtype mice or mice that had genome-wide uPAR deficiency. Homozygous uPAR knockout mice were bred on a B6 background; uPAR knockout fibrosarcoma cells were derived from the B6 MC17 sarcoma cell line using CRISPR-Cas editing. Litters of wildtype and knockout mice were randomized at 5 weeks of age into 12 groups, with a total of 43 mice included in the experiment. The intent was to treat mice starting at day 16 after tumor inoculation, once tumors were grossly palpable (>0.5 cm in diameter). In total, the experiment included 11 wildtype female mice, 9 wildtype male mice, 10 uPAR knockout female mice, and 13 uPAR knockout male mice. Fifteen mice were assigned to the group receiving vehicle control (saline), thirteen mice were assigned to the group receiving eBAT (50 µg/kg in two weekly cycles), and fifteen mice were assigned to the group receiving meBAT (50 µg/kg in two weekly cycles). Mice were sacrificed when they reached a tumor endpoint (tumor volume of 1.5 cm^3^, evidence of ulceration, or tumor-related ill-thrift). 

Gross necropsies were conducted in all 43 mice at the time of death; samples from twenty-five mice were examined histologically. Tumor-related adverse events occurred, including tumor invasion into the muscle and/or the abdominal wall. Tumors growing inside the body cavity were associated with severe pathology, including diffuse organ infiltration and severe internal hemorrhage.

As was true in the experiments described above, there were no physical adverse events associated with administration of eBAT or meBAT to wildtype B6 mice or uPAR knockout B6 mice. Histologically, both were associated with an incidental increase in myelopoiesis. Although no liver pathology was detected in any of the mice examined, we cannot rule out that these lesions could occur at a low frequency in the B6 strain. Taken together with the other data we report, our findings indicate that the presence or absence of a uPAR sink does not have bearing on the occurrence of toxicity associated with these compounds. 

### 2.5. The Effect of eBAT on Dogs with Hemangiosarcoma

The observed safety of eBAT (and meBAT) in mice, along with previous evidence of activity against various tumors in vitro and in vivo [2,3,28,29,30], supported additional assessment of eBAT in larger animals, which would complement the mouse data and enhance the relevance to human applications.

Three independent clinical studies, two for which the efficacy date have been previously reported [4,31], were designed in dogs with spontaneous hemangiosarcoma and are described in detail in the Materials and Methods Section. While the methods, purpose of studies, and end results for efficacy differed across the three studies, the adverse events are cumulatively addressed for the first time in this report, summarizing the toxicity profile of eBAT in dogs with naturally occurring hemangiosarcoma. Although discussed cumulatively below, Supplemental Table S1 shows the occurrence of adverse events in relation to dose and the individual in which they occurred to provide full context and clarity of these results.

The first experimental study (SRCBST-1) enrolled 23 dogs with histologically confirmed stage 1/2 spontaneously occurring splenic hemangiosarcoma and ECOG performance status scores between 0 and 2 [4]. Dogs in this experiment received a single cycle of eBAT (three treatments over a 1-week period on an MWF schedule) after they had surgery to remove their primary tumor and before starting adjuvant chemotherapy. The biologically active dose of 50 µg/kg was derived from this adaptive trial, and it was selected as the dose that was used for the experiments described in B6 and BNX mice above. The second experimental study (SRCBST-2) enrolled 26 subjects with advanced splenic hemangiosarcoma, including 1 dog with confirmed regional metastasis and 5 dogs that presented to their veterinarian with ruptured tumors and abdominal hemorrhage (confirmed stage 2) as well as evidence of liver nodules. These dogs received three weekly cycles of eBAT (50 µg/kg) after surgery immediately preceding the first round, and prior to the third and fifth round of doxorubicin chemotherapy [31]. The third experimental study (eBAT-CC) was designed to evaluate safety of this drug in dogs with hemangiosarcoma independent of stage or anatomic location. It consisted of 29 dogs that received at least one dose of eBAT (50 µg/kg). All of the dogs were treated with surgery to excise the primary tumor. Ten dogs had no gross disease at screening; residual or recurrent gross disease was present at the time of eBAT administration in nineteen dogs. Three dogs had one solitary nodule or mass. Nine dogs had multiple discrete nodules or masses, and six dogs had too-numerous-to-count nodules or masses. Chemotherapy was administered prior to eBAT in 18 dogs and following eBAT in 10 dogs. 

Of the 78 dogs included in these combined studies, 23 dogs (29%) experienced at least one adverse event, although all of the adverse events recorded were mild to tolerable (grade 3 or lower; Table 2). Of the 78 dogs, 8 (10.2%) experienced elevated liver enzymes after eBAT administration. There were four grade 1 ALT elevations, three grade 2 ALT elevations, and three grade 3 ALT elevations. There were two grade 1 ALP elevations, three grade 2 AST elevations, and two grade 3 AST elevations. For all of the dogs where follow-up laboratory analyses were available, the hepatic toxicities were fully reversible.

Of the 78 dogs, 12 (15.3%) experienced collapse or a hypotensive event during the administration of eBAT or in the immediate post-injection period. In 11 of these cases, the events occurred upon repeated dosing of eBAT: one event occurred during the second eBAT treatment, six events occurred during the third eBAT treatment, and four occurred during or after the fourth eBAT treatment. All of the hypotensive events were considered to be grade 2 and immediately responded to fluid therapy. Of the three dogs in the first experimental study that received 100 µg/kg (higher than the biologically active dose), two experienced collapses. It should be noted that two of the included hypotensive events were associated with what was consistent with anaphylactic infusion reactions during the fourth eBAT treatment in the second experimental study. The observed symptoms in these dogs included hypotension, vomiting, diarrhea, erythema, and swelling. In both cases, the symptoms were immediately responsive to anti-nausea medication, fluid therapy, diphenhydramine, and corticosteroids.

Of the 78 dogs, 2 (2.6%) experienced seizures. Neither dog had a prior history of seizures. The first dog, enrolled in the first experimental study, had a petit seizure that lasted about 30 s during the first eBAT treatment. Drug administration was discontinued, and the dog recovered uneventfully and returned to complete the subsequent two treatments of the cycle without further evidence of adverse events. The second dog, enrolled in the second experimental study, experienced a seizure during the fourth eBAT treatment. This dog also recovered uneventfully and proceeded to receive five additional eBAT doses without complications.

Of the 78 dogs, 8 (10.2%) experienced gastrointestinal toxicity during eBAT administration, consisting of nausea and vomiting. All dogs were treated with intravenous maropitant. In all cases except one, the nausea occurred after repeated eBAT administrations.

Eleven of the seventy-eight dogs, including three from the first experimental study, six from the second experimental study, and two from the third experimental study, had full necropsies performed at the Veterinary Diagnostic Laboratory of the University of Minnesota (D-Lab, *n* = 10) or at the North Carolina State University College of Veterinary Medicine (*n* = 1).

The dogs that were necropsied ranged in age from 5 to 14 and had received their first dose of eBAT 31 to 453 days before they died. Eight dogs died from progressive disease with disseminated metastasis to various organs (heart, liver, and omentum). Changes commonly seen as a sequela of age-related organ failure in dogs, including myocardial degeneration, endocardiosis, ischemic renal infarcts, interstitial nephritis, etc., were seen in varying degrees for all of the dogs. All eight of these dogs had a terminal event (hemorrhage) that precipitated their natural death or euthanasia. Three dogs died with no evidence of hemangiosarcoma. One of these dogs (a 5-year-old) that had received eBAT 161 days prior to death, died from cardiomyopathy, which might have been age-related or secondary to doxorubicin chemotherapy. Another dog (a 14-year-old) had end-stage kidney disease, valvular degeneration, and myocardial fibrosis, all of which were considered to be age-related. This dog had received eBAT 453 days prior to death and was considered among the exceptional survivors. Finally, one dog (a 10-year-old) had right-sided heart failure and renal tubular necrosis, suggestive of a recent hypoxic/ischemic event as can occur with heart disease. The dog had received two doses of doxorubicin prior to eBAT; so, while no overt cause for an unexpected death was appreciated, a myocardial event was a consideration. However, other causes such as biochemical disturbances secondary to the dog’s treatment could not be ruled out. This dog had received eBAT 31 days prior to death. A summary of necropsy results can be found in Supplementary Table S2.

In summary, these three experimental clinical studies in tumor-bearing dogs show remarkable consistency, with manageable (<grade 3) adverse events noted, and with most being reversible or easily controlled with medication (e.g., prophylactic intravenous fluids to support blood volume and prevent hypotension and maropitant to counter or prevent nausea and vomiting).

## 3. Discussion

eBAT is a unique drug developed at the University of Minnesota (and currently licensed to Anivive Lifesciences, Inc. for use in all non-human animals), so there is no independent history of its toxicological profile beyond this report and the data on file with the U.S. Food and Drug Administration. Thus, here, we detail for the first-time toxicities associated with eBAT administration in healthy mice, tumor-bearing mice, and dogs with hemangiosarcoma. Because part of the predicted mechanism of action of eBAT involves elimination of cells that express EGFR, we predicted its toxicity profile might be similar to those of other EGFR-targeted therapies. Significant toxicities were not observed in our study subjects, and we noted that eBAT was extremely well tolerated at doses resulting in biological activity.

eBAT has dual specificity and we believed this would confer greater efficacy and lower toxicity compared to monospecific targeted drugs. In an earlier study, we observed that eBAT was less toxic than its monospecific counterparts, EGF-PE and uPA-PE [4]. Additionally, the toxicity of other EGFR targeted therapies such as erlotinib and gefitinib is related to signal-triggering and tyrosine kinase inhibition resulting in xerosis, pruritus, alopecia, and hand and foot reactions, among others [32,33]. The mechanism of action of eBAT is quite different. Immunotoxin complexes bind specifically to their targeted receptors and are then endocytosed, enter the cytoplasm, and access the endoplasmic reticulum where they catastrophically act against cellular protein translation (specifically elongation factor 2) [3,5]. It is believed that failure to bind their targeted receptor results in off-target binding and pinocytosis, leading to adverse events. We believe that the lack of interference with the EGFR signaling pathway may be in part responsible for the reduced toxicity of eBAT. 

Herein, we document that eBAT was associated with predictable and relatively mild adverse events at biologically active doses. At higher doses, administration of eBAT to CD1 mice resulted in dose-dependent increases in hepatocellular damage, based both on the release of liver enzymes into the circulation and the hepatic injury documented by histopathology observed at doses higher than the biologically active dose of 50 µg/kg [4]. Of note, these same hepatic histopathologic changes were not seen at the lower doses in CD1 mice, and they were not consistently seen in B6 or BNX mice. 

Approximately 10% of the dogs receiving eBAT experienced gastrointestinal adverse events involving nausea, inappetence, or diarrhea. All of the cases were mild and did not require dose reductions or dose delays. In all cases, the nausea symptoms resolved with intravenous maropitant. Other adverse events that occurred in the dogs in our studies were hypotensive collapsing episodes and seizures in about 15% and 2% of the subjects, respectively. Hypotension and vascular leak syndrome are commonly reported in clinical studies of other immunotoxins containing PE [34]. For example, hypotension was reported in a previous study investigating treatment of advanced solid tumors with immunotoxin LMB-1 [35] as well as refractory B-cell malignancies treated with the bispecific ligand-directed toxin DT2219 [36]. The pathophysiology of hypotension from eBAT is not completely understood, but it is possible that this side effect may be due to endothelial cell endocytosis leading to capillary leak syndrome [37,38]. In the human clinical trials cited above, capillary leak syndrome was determined to be dose-dependent and a concern at higher drug concentrations. In the dogs in our studies, all of our collapsing events responded rapidly to fluid boluses. This side effect was manageable, fully reversible, and potentially not related to the dose of eBAT administered.

meBAT was developed to address the possibility of low affinity uPAR binding to its receptor in mice. In our studies, no physical or histopathological adverse effects could be attributed to meBAT administration. Similar results have been found in two additional studies (our unpublished data), suggesting the tolerability of meBAT is reproducible.

The use of companion animals with spontaneously arising tumors [39,40,41,42] allowed for investigations into the safety, efficacy, and mechanisms of action of eBAT in a setting that would realistically model its use for human cancer patients. As these were client-owned dogs with cancer, the majority of toxicity data were derived from hematologic, biochemical, and physiologic diagnostics and observations. The mild hepatotoxicity was corroborated in canine studies, as 10.2% of the treated dogs had asymptomatic elevations in liver enzymes. Importantly, the liver toxicity was transient, self-limiting, and reversible, with no evidence of chronic eBAT-associated changes in liver histology seen in the liver of any of the dogs that were necropsied weeks to months after treatment. Despite its tolerability, these studies suggest that using clinical chemistry to monitor liver injury during eBAT administration would be prudent.

As noted above, dogs were companions and family members, so neither euthanasia nor necropsy were study endpoints. Necropsy was encouraged, and 14% of owners elected to have full necropsies when their dogs died. The deaths occurred weeks to months to years after eBAT administration, so the only changes that could have been detected would have been those that led to chronic or permanent gross or microscopic pathology. The findings from the necropsies were variable with many dogs succumbing to metastatic hemangiosarcoma or other chronic comorbidities. Importantly, there was no evidence of chronic or permanent damage to any tissue on necropsy that could be attributed to eBAT. We should note that the CNS was not assessed in the majority of the dogs, although there were no behavioral changes to support any CNS pathology.

At the present time, we have only investigated the therapeutic use of eBAT in dogs with hemangiosarcoma. This is a uniformly fatal cancer of dogs where a diagnosis is often not made until the disease is very advanced, and few drugs have demonstrated any clinical benefit. eBAT has the potential to prolong the lifespan of these dogs [4], and our studies support that the drug is safe, with a low toxicity profile. Owners of dogs with cancer are often willing to tolerate low-grade toxicities if anticancer treatments can provide long-term benefit. Furthermore, we are exploring the use of eBAT in the treatment of healthy dogs that do not have a cancer diagnosis but have been deemed to be at high risk for cancer development based on a novel test for early detection and risk assessment [43].

In conclusion, the totality of our results warrants continued exploration of eBAT as a therapeutic agent for humans and dogs with cancer. Our previously published efficacy studies in dogs, as well as the relatively modest toxicities reported here, indicate that treatment mostly will be tolerable and result in no, minimal, or acceptable changes in quality of life. Future studies will be designed to focus on the mechanisms of eBAT as it pertains to angiogenesis, tumor inflammation, and other components of the tumor microenvironment.

## 4. Materials and Methods

### 4.1. eBAT and meBAT

The test articles, human eBAT and the comparable meBAT mouse homolog, were manufactured using a bacterial inclusion body system as described [2]. Both drugs were tested for potency and sterility before they were used in live animals. Release criteria for both drugs were established regarding drug purity (>95%), endotoxin (<50 Eu/mg), in vitro potency (IC50 < 1.0 nM), sterility, and concentration prior to use in live animals. Stability and potency were confirmed by retesting the drugs’ killing activity in vitro at 90–180 month intervals, and/or prior to each experiment.

### 4.2. Cell Lines

The cell lines used in this study included the DHSA-1426 canine hemangiosarcoma line, used for xenograft experiments. Mouse fibrosarcoma MC17 cells (B6, H2^b^ background) were obtained from ATCC for syngeneic experiments. Targeted inactivation of the uPAR gene in MC17 cells was carried out using clustered regularly interspaced short palindromic repeat (CRISPR) gene editing by Applied Biological Materials Inc. (ABM, Richmond BC, Canada). Exon 2 of the mouse uPAR gene was targeted to insert a guanine (G) base at position 509, shifting the reading frame. Two mixed derivatives (K02 and K06) were generated by ABM. Clonal sublines (H6 and D10) were obtained by limiting dilution. The presence of the targeted insertion in the lines was confirmed by Sanger sequencing and the absence of surface expression of mature uPARs was confirmed by flow cytometry. Cell line authentication was performed on a pre-set schedule according to established standard operating procedures in the laboratory of JFM. The DHSA-1426 and the MC-17 and D10 cell lines were confirmed to be of the correct species (dog and mouse, respectively) and to be devoid of other mammalian contaminants by microsatellite testing (single tandem repeat panels) by IDEXX BioAnalytics (North Grafton, MA, USA). Cell lines were also documented to be free of contamination by any *Mycoplasma* sp. based on polymerase chain reaction (PCR) testing at IDEXX BioAnalytics. The DHSA-1426 cell line is available through Kerafast, Inc., (Boston, MA USA).

### 4.3. Mice

CD-1 mice aged 4 to 5 weeks used for an eBAT toxicity study were obtained from Envigo. C57Bl/6 (B6) mice aged 5 to 7 weeks used for the meBAT toxicity study were obtained from the Jackson Laboratories. uPA receptor (uPAR) knockout (KO) mice used to examine how uPAR expression in tumor cells and in the microenvironment impacted the safety and efficacy of eBAT and meBAT were also obtained from the Jackson Laboratories as two heterozygous breeding pairs. Wildtype and uPAR KO mice were bred in our own colony. The homozygous uPAR KO mice and the uPAR wildtype mice derived from these breedings were phenotypically indistinguishable, both in the mixed litters (bred from heterozygous parents) and in the subsequent homozygous litters. The uPAR KO mice were fertile, and bred with equivalent frequency, had litters of approximately the same size, and showed no greater perinatal mortality than the uPAR wildtype mice. Beige-nude-XID mice aged 5 to 7 weeks used for the eBAT and meBAT toxicity studies in animals harboring tumor xenografts were obtained from Charles River Laboratories. For all experiments, animals were examined and weighed. All animal studies were conducted with approval and under the oversight of the University of Minnesota institutional IACUC (protocol numbers 1609-34115A, 1601-33435A, and 1812-36575A).

### 4.4. Dogs

All of the dogs included in this report were companion animals that participated in one of three independent IACUC-approved clinical studies evaluating the safety and efficacy of eBAT with owner consent (protocol numbers 1110A06186, 1507-32804A, 1508-32969A, 1812-36570A, 1807-36169A, and 2110-39474A). Dogs in the three studies had a confirmed histologic diagnosis of hemangiosarcoma and met the relevant eligibility criteria for the studies. The eligibility criteria for the Sarcoma Bispecific Toxin studies (SRCBST-1 and SRCBST-2) have been described previously [4,31]. In order to participate in the third study, called eBAT-Compassionate Care (eBAT-CC), dogs needed to have a definitively diagnosed (histopathology) and staged (imaging) hemangiosarcoma (HSA) of any visceral site, but not the skin, ECOG performance status of 0 or 1, hematocrit >22%, and no severe comorbidities (e.g., advanced kidney, liver or heart disease, or coagulopathies). Dogs were not eligible if they were receiving any concurrent cytotoxic drugs. For dogs that received cytotoxic chemotherapy, a 14-day washout was required previous to enrollment. Dogs were also not eligible if they were receiving supplements or alternative medications. For dogs receiving supplements or alternative medications, a 5-day washout was required previous to enrollment. Concurrent use of prednisone and/or nonsteroidal anti-inflammatory drugs (NSAIDS) was allowed [44,45,46].

### 4.5. Non-GLP eBAT Toxicology Study in Healthy Mice

A formal dose-ranging toxicology study including four groups of twelve CD-1 mice (six males and six females) administered four escalating doses of eBAT and a saline control group (six males and six females) was carried out at the University of Minnesota Center for Translational Medicine (CTM) testing facility. This study was conducted using good laboratory practice (GLP) guidelines (https://www.fda.gov/media/165993/download, accessed on 1 April 2024). The CD-1 outbred strain was chosen (*n* = 60) because it is commonly used for toxicology studies. 

The sample size of this experiment was designed primarily to detect liver injury, with the liver being the most likely (and most sensitive) target organ based on previous experience with EGFR-targeted therapies. We used the accepted reference intervals for liver enzymes for this strain, assumed the analyses would use a *t*-test on log-transformed data (because of skewed data and higher variability at higher doses), and reported the most conservative power across the four combinations of AST/ALT and male/female:

With 6 animals in each group (separating sex), we expected to have 80% power to detect an increase in the mean of the ALT and AST concentrations as small as 110% of the maximum of the reference intervals. In other words, the experiment would detect an elevation of 1.1-fold in the concentration of liver enzymes in serum. Four animals per group would have provided 80% power to detect an increase in the mean to 135% of the maximum of the reference intervals.

The dosing of eBAT included groups administered 40 μg/kg, 80 μg/kg, 120 μg/kg, and 200 μg/kg through the intravenous route. Normal saline solution (0.9% sodium chloride) was used for the vehicle control group. Mice received eBAT in two weekly cycles on Monday, Wednesday, and Friday intravenously into the lateral tail vein. The experiment was terminated after the final treatment for cycle 2 on day 13.

The following parameters and endpoints were evaluated in this study: survival, body weight, cage side observations, hematology and clinical chemistry parameters, and select tissues collected at necropsy for histology and review by a board-certified veterinary pathologist (JTS). Liver, spleen, kidney, pancreas, lung, stomach, small intestine, and heart were collected for histopathology. Samples were fixed in 10% neutral buffered formalin and routinely processed, and slides were stained with H&E according to standard methods.

### 4.6. meBAT Toxicology Study in Healthy Mice

We surmised that the safety of eBAT seen in preliminary studies [4] and in the non-GLP mouse study might be due to the lower affinity of human uPA for mouse uPAR, possibly sparing inflammatory, vascular, and connective tissue cells from the effect of the toxin. To investigate this, we synthesized a mouse-specific eBAT (meBAT) to evaluate the toxicity of dual targeting of EGFR and uPAR with a BLT in vivo. To make meBAT, we replaced the human ATF of uPA with the mouse ATF of uPA and showed that meBAT binds mouse sarcoma cells with high affinity and is cytotoxic against mouse osteosarcoma cells at nanomolar concentrations [47]. The toxicity study was conducted using three groups of eight (four males and four females) B6 mice administered three escalating doses of meBAT, and a saline control group (one male and one female). The total number of animals in this study was 26. The B6 strain was chosen because it represents an immunocompetent inbred strain that is syngeneic to many tumors where mechanisms of action can be evaluated.

Having baseline expectations from the CD-1 experiment (consistent elevations in liver enzymes >2-fold and histological changes) and considering the power to detect liver injury, we determined groups of 3–4 mice were sufficient for the experiments described in this and the following sections that utilized B6 and BNX mice.

The dosing of meBAT included groups administered 50 µg/kg, 100 µg/kg, and 200 µg/kg through the intraperitoneal route. Mice received meBAT in two weekly MWF cycles. The experiment was terminated after the final treatment for cycle 2 on day 15. Gross necropsies were conducted and samples from liver, lungs, kidneys, adrenals, esophagus, heart, small intestine, pancreas, spleen, stomach, and omentum were examined microscopically in two representative mice from each group (saline control, 50 µg/kg, 100 µg/kg, and 200 µg/kg).

### 4.7. eBAT and meBAT Toxicology Study in Mice Harboring Canine Hemangiosarcoma Xenografts

To examine the effects of eBAT in animals with hemangiosarcoma, three groups of four immunodeficient, female beige-nude-XID (BNX) mice received a subcutaneous injection of DHSA-1426 canine hemangiosarcoma cells embedded in Matrigel as described [14]. The total number of animals in this study was 12. The BNX strain was chosen because it is permissive for tumor xenografts. The dosing of eBAT and meBAT for treated groups was 50 µg/kg through the intraperitoneal route. The mice were treated with one cycle of eBAT or meBAT administered on day 3, day 6, and day 9 after tumor inoculation. One group received saline as a control. Four mice died prematurely and were necropsied and evaluated for eBAT toxicity. Gross pathology and histopathology were performed in an additional four mice at the experimental endpoint of 40 days.

### 4.8. eBAT and meBAT Toxicology Study in Wildtype Mice and uPAR KO Mice Harboring Syngeneic Sarcomas

This experiment included a total of 43 B6 mice. There were 11 wildtype female mice, nine wildtype male mice, 10 female uPAR KO mice, and 13 male uPAR KO mice. In each group, mice were sequentially assigned to receive SQ injections of MC-17 fibrosarcoma cells or D10 cells and treatment with eBAT (50 µmg/kg), meBAT (50 µg/kg), or saline control (Table 3). eBAT and meBAT were administered IP in two weekly cycles starting at day 16, after tumors were grossly measurable. The endpoint for the experiment was day 60. Gross necropsies were conducted in all of the mice, and samples from twenty-five mice were examined histologically.

### 4.9. Statistical Analysis

GraphPad PRISM version 8.3.1 (GraphPad Prism Software, Inc., La Jolla, CA, USA) was used to create statistical tests. All multiple comparison studies used one-way ANOVA with repeated measures, while single comparison studies used Student *t* test.

### 4.10. Canine Experimental Clinical Study 1 (SRCBST-1)

The parameters of this trial have been described previously [4]. eBAT was administered in the adjuvant setting at least 14 days after surgery as one cycle of three treatments (WFM) prior to standard doxorubicin chemotherapy protocol. Doxorubicin was administered starting no sooner than 14 days after the last eBAT treatment. No supplements or other drugs were allowed until the dog showed disease progression. Briefly, this study followed a Bayesian adaptive phase I–II trial design to evaluate efficacy and toxicity. Eligibility criteria included dogs with histologically confirmed stage 1/2 spontaneously occurring splenic hemangiosarcoma and a good ECOG performance status score (0–2). Dose-finding was determined based on predefined criteria of acceptable toxicity (no dose-limiting AEs) and efficacy (>50% survival at 6 months) to jointly model toxicity and efficacy [48]. The planned dose escalation started with cohorts of three dogs receiving eBAT intravenously at 25 µg/kg, 50 µg/kg, and 100 µg/kg. Treatments consisted of one cycle (three treatments of eBAT on WFM). Results were compared to a contemporary cohort of dogs with stage 1/2 splenic hemangiosarcoma treated with the standard of care at the University of Minnesota Veterinary Medical Center (VMC). Cumulative analyses of toxicity and signals of efficacy were carried out after each dog was enrolled and at the 6-month anniversary of enrollment. This study was terminated after the 23rd dog was enrolled because it met the goals of safety and efficacy. Adverse events were graded based on Veterinary Cooperative Oncology Group—Common Terminology Criteria for Adverse Events (VCOG-CTCAE v1) guidelines [49]. Necropsy at the time of death was encouraged. Three dogs from SRCBST-1 had full necropsies done at the Veterinary Diagnostic Laboratory of Minnesota.

### 4.11. Canine Experimental Clinical Study 2 (SRCBST-2)

The parameters of this trial have also been described previously [31]. Briefly, this study was initiated with the enrollment of the 24th consecutive dog entering study 1 with identical eligibility criteria. Three variables were modified: (1) dosing was increased to three cycles of three treatments each, with the first cycle given after surgery on days 1, 3, and 5 of the week prior to the first doxorubicin administration, the second cycle given immediately prior to the second chemotherapy cycle (day 24), and the third cycle given immediately prior to the fifth and final chemotherapy cycle (day 88); (2) chemotherapy was started the day after completion of each eBAT cycle to simplify visits for pet owners; (3) dogs with advanced-stage disease (stage 3) were eligible if they had metastasis in the abdomen and if the metastatic lesions were resectable. Full necropsies were performed for six dogs in the SRCBST-2 study. 

### 4.12. Canine Study 3 (eBAT-CC)

This study was designed as a compassionate care option for dogs with hemangiosarcoma. Dogs were eligible to enroll with any stage of disease (including with gross metastatic burden). The main goal was to obtain additional data to understand potential toxicity associated with systemic eBAT administration. A single cycle (three treatments of eBAT on WFM) could be administered before or after systemic chemotherapy. When given after chemotherapy, the last cycle had to be completed at least 14 days prior to eBAT. When given before chemotherapy, the first cycle could not start for at least 14 days after eBAT.

## Figures and Tables

**Table 1:** Dose-dependent biochemical and histopathological findings from non-GLP mouse study.

Group			
µg/kg	Biochemical	Anatomic Liver	Anatomic GI
Saline	None	None	None
40	None	(Periportal hepatocellular microvesicular vacuolar change and hepatocellular regeneration)	None
80	None		None
140	Dose-dependent increase in hepatocellular enzymes	(Dose-dependent portal biliary hyperplasia, lymphoplasmacytic inflammation, periportal microvesicular change, hemorrhage, coagulation, necrosis, and apoptosis)	None
200	dose dependent changes in several parameters of liver function (hypoglycemia, hypoalbuminemia, and hyperbilirubinemia)		(Villous atrophy and submucosal edema)

**Table 2:** Number of dogs experiencing elevated liver enzymes.

Grade	ALT	ALP	AST
1	4	2	0
2	3	0	3
3	3	0	2
4	0	0	0

**Table 3:** Groups for experiment evaluating the impact of uPAR expression.

Group	Mouse Strain	Subcutaneous Injection	Drug/Dose
1	Wildtype	MC-17 fibrosarcoma cells	eBAT (50 µg/kg)
2	Wildtype	MC-17 fibrosarcoma cells	meBAT (50 µg/kg)
3	Wildtype	MC-17 fibrosarcoma cells	Saline
4	uPAR-KO	D10 uPAR-KO cells	eBAT (50 µg/kg)
5	uPAR-KO	D10 uPAR-KO cells	meBAT (50 µg/kg)
6	uPAR-KO	D10 uPAR-KO cells	Saline

## Data Availability

Data from the canine clinical studies are unavailable due to owner privacy issue restrictions.

## Supplementary Materials

The following supporting information can be downloaded at: https://www.mdpi.com/article/10.3390/toxins16090376/s1, Figure S1: BNX mouse weight data; Table S1: Data summary for each individual dog; Table S2: Canine necropsy results.
