# Supplementary material for: Toxicity Profile of eBAT, a Bispecific Ligand-Targeted Toxin Directed to EGFR and uPAR, in Mice and a Clinical Dog Model
Source: Toxins (Basel). 2024 Aug 26;16(9):376. doi: 10.3390/toxins16090376 (PMC11436214; doi:10.3390/toxins16090376)
Supplement: Supplementary file 1 [file toxins-16-00376-s001.zip › Figure S1.pdf]

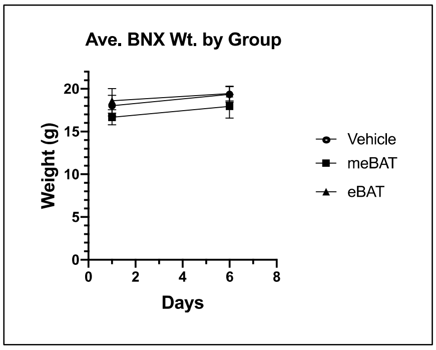

Supplemental Figure S1. BNX mice given DHSA 1426 hematosarcoma cells were treated with 50 ug/kg eBAT or 50 ug/kg meBAT on MWF. Day 0 was the first injection. Mice were weighed on day 1 and day 6. Statistical analysis revealed no differences between any of the groups.
