# Supplementary material for: Toxicity Profile of eBAT, a Bispecific Ligand-Targeted Toxin Directed to EGFR and uPAR, in Mice and a Clinical Dog Model
Source: Toxins (Basel). 2024 Aug 26;16(9):376. doi: 10.3390/toxins16090376 (PMC11436214; doi:10.3390/toxins16090376)
Supplement: Supplementary file 1 [file toxins-16-00376-s001.zip › Table S2.pdf]

Supplemental Table S2. Canine necropsy results.

**Group**	**Tissue**					
	**Liver**	**GI**	**Kidney**	**Lung**	**Heart**	**Other**
SRCBST-1	Multifocal HSA nodules	Jejunal HSA mass	Cortical atrophy and fibrosis, medullary fibrosis (infarction), focally extensive, marked, chronic glomerulosclerosis and tubular sclerosis	Multifocal HSA metastses	HSA mass	Hemoabdomen; Liver, lung, small intestine (jejunum), heart, diaphragm - HSA
SRCBST-1	Metastatic HSA, multifocal and ruptured masses; Left lateral liver lobe, right medial liver lobe – capsular fibrosis, multifocal, mild, chronic	None	Lymphoplasmacytic interstitial nephritis, multifocal, mild, chronic	None	Right auricle/atrium – HSA, locally extensive; Mitral valve - endocardiosis, multifocal, mild to moderate, chronic	Hemoabdomen; Omentum, broad ligament, prostate – metastatic HSA
SRCBST-1	Liver mass - HSA	Jejunum: hemorrhagic enteritis, multifocal, mild.	None	None	Multifocal HSA ; myxomatous valvular degeneration (endocardiosis), multifocal, mild.	Hemoabdomen; Omentum, mesentery, liver, right auricle, diaphragm: HSA, metastatic.

SRCBST-2	Multifocal masses -HSA	None	Cortical atrophy and fibrosis, multifocal, mild,	None	Right auricle HSA; myxomatous valvular degeneration, tricuspid and mitral valves, locally extensive, moderate, chronic [endocardiosis]	Hemoabdomen; Liver, mesentery, diaphragm, heart (right auricle) – HSA; –nodular cortical hyperplasia, bilateral, diffuse, mild, chronic
SRCBST-2	Hemangiosarcoma, multifocal; Parenchymal cysts, multifocal, random, marked, chronic, with multifocal rupture.	None	None	None	Myxomatous valvular degeneration (endocardiosis), multifocal, mild, chronic.	Marked ascites, mild hydrothorax
SRCBST-2	Multifocal HSA				Right atrial hemangiosarcoma, Ventricular septal defect, high membranous, non-patent, focal, moderate, chronic<br>b. Mitral valve: Myxomatous valvular degeneration (Endocardiosis), coalescing, mild to moderate, chronic	Hemoabdomen

SRCBST-2			Nephritis, suppurative, multifocal, marked with intralesional bacteria; cortical fibrosis, multifocal, marked, chronic with obsolescent glomeruli; interstitial nephritis, lymphoplasmacytic, multifocal, mild, chronic; congestion, widespread, moderate, acute	pulmonary edema and congestion	Left and right ventricles, cardiomyocyte degeneration with ventricular chamber dilation and decreased ventricular free wall thickness (DCM ; myocardium, fibrosis, multifocal to coalescing, marked, chronic; mitral valve, myxomatous valvular degeneration (endocardiosis), multifocal, mild, chronic; tricuspid valve (septal leaflet), myxomatous valvular degeneration (endocardiosis), mild, chronic	death due to heart disease
SRCBST-2	multifocal masses: HSA				right auricular mass-HSA	hemoabdomen; Right auricle, omentum, liver: HSA
SRCBST-2	multifocal nodules HSA		cortical atrophy and fibrosis, multifocal, mild, chronic.		myxomatous valvular degeneration (endocardiosis),	hemoabdomen; HSA liver, omentum
eBAT-cc	Biliary adenoma, focal; hepatocellular nodular hyperplasia, multifocal, mild to moderate, chronic; lipogranulomas, multifocal, moderate, chronic	Leiomyoma, focal	Cortical atrophy and fibrosis, widely-disseminated, marked, chronic; tubular degeneration/necrosis, atrophy, loss, and ectasia, widely-disseminated, moderate, chronic with mild regeneration; glomerulosclerosis and obsolescence, widely-disseminated, moderate to marked, chronic; interstitial		interstitial fibrosis, multifocal, moderate, chronic with cardiomyocyte degeneration/loss and mild fatty infiltration; left and right atrioventricular valves – myxomatous valvular degeneration (endocardiosis), multifocal,	NED, "end-stage kidneys"

			nephritis, lymphoplasmacytic, widely-disseminated, mild to moderate, chronic			
eBAT-cc	Marked hepatic cord atrophy with centrilobular fibrosis (consistent with chronic passive congestion; Focal fibrous plaque (presumed) (gross diagnosis)		Moderate, multifocal tubular necrosis with regeneration; Chronic, multifocal renal infarcts (gross diagnosis)	Focal osseous metaplasia; Mild, multifocal, pneumoconiosis	Moderate multifocal adipocyte infiltration with mild perivascular fibrosis and cardiomyocyte vacuolation b) Mild mitral valve endocardiosis	Death due to myocardial event prioritized, NED HSA

HAS – Hemangiosarcoma
NED- No evidence of Disease
